# A farewell from the “old” Editor-in-Chief

**DOI:** 10.1007/s11547-020-01325-5

**Published:** 2021-01-28

**Authors:** Andrea Giovagnoni

**Affiliations:** 1 Department of Clinical, Special and Dental Sciences, University Politecnica delle Marche, Ancona, Italy; 2 Department of Radiology, University Hospital “Umberto I – Lancisi – Salesi, Via Conca 71, 60126 Ancona, Italy

Dear Readers,

I reach the end of my term as Editor-in-Chief of *“La Radiologia Medica”.*

I am leaving my position not without a hint of nostalgia, but also with the awareness and the pride for having contributed to the growth of the SIRM official journal, turning it into a reputable, international scientific journal that reached the psychologically unbreakable Impact Factor score of 2, never been so high in its more than centenary life.

Eight years have passed since, at the end of October 2012, the then-President Carlo Faletti proposed to me to become Editor in-Chief of “La Radiologia Medica”, the official SIRM Scientific Journal.

The President’s mandate was clear in its harsh and essential simplicity: the aims were to renew the magazine while preserving its tradition, to avoid financial and—mostly—editorial collapses, to increase international credibility, and to retain Members.

At that time, the Journal was going through a difficult period: the number of manuscripts received by the editorial office was at a worrying historical minimum peak; a bilingual journal (Italian and English) printed and sent by mail in hard copy to all the SIRM Members was showing all the signs of the time and needed a deep renew.

I proudly accepted the assignment with the serene concern of being embarking on a long and tough journey; in a short time, a new editorial line was defined on few but substantial characterizing points: 12 issues per year (instead of 8), a cover restyling, an online-only and English-only journal with abolition of hard copies; finally, a renovation of the revision process by improving the number and the selection of the reviewers, and a more attentive selection of the manuscripts on the basis of strict quality parameters.

The first issue of the *new edition*, renewed in the graphics and in the content, was regularly published online in January 2013 in English language.

Since then, the Journal, which became monthly, had a steady growth trend, with a double-digit percentage of increases both for the number of submitted manuscripts and as well as for the number of downloads per year.

At present, the editorial office of “La Radiologia Medica” manages more than 1000 manuscripts yearly submitted from about twenty countries around the world.

In few years, the acceptance rate dropped from 42 to 22%, this in contrast with a significant increase of 43% in the number of the published papers, for a total of 228 papers published per year.

The number of the invited reviewers increased from a few dozens to over 460, guaranteeing a faster and more transparent reviewing process including a wide range of skills, with a consolidated, average of 2 weeks for the completion of the review process.

A great satisfaction has been the constant increase of the IF which reached the value of 2, one of the highest among the radiological journals of the National Scientific Society, and with a self-citation rate significantly below 20%.

Following this encouraging performance, the Journal strongly consolidated itself as a pivotal asset of the Society, establishing itself as the “SIRM-gold card”, and became at the same time the object and the active subject of stimulating discussions during the Institutional meetings, which were raised in number and effectiveness, that the successive SIRM Presidents held with the managers of other International Scientific Societies.

These important and consolidated goals are the result of careful planning and of a believed and effective teamwork that involved the entire Editorial Board of the Journal, the office and the Springer editorial managers, irreplaceable for the great and punctual technical support, and the SIRM Presidents *Carlo Faletti*, *Carlo Masciocchi*, *Carmelo Privitera* and *Roberto Grassi* who supported my efforts in the management of the Journal: my personal endless thanks go to all of them.

After 8 years in the role, at the end of my experience as Editor, I felt that the time to throw the “ball” to a new Direction, younger, more motivated, more capable of grasping the new challenges of scientific publishing: Open access, economic management independence, new direction and editorial experiences open to internationalization.

The choice of *Prof. Antonio Barile* as the new Editor in-Chief, who supported me managing the Journal in the last two years, was conceived in this direction, as the natural continuum of a project begun in 2012 that aims to grow and bring “La Radiologia Medica” at even more ambitious levels.

*Antonio Barile* is the right man at the right time, capable, determined, now master of the fine mechanisms of the scientific publishing world.

Good job *Antonio*: I’m sure I am leaving the Journal in the best hands possible at now.

I am saying this already as “Old” Editor in-Chief of the Journal, and I am saying that to you like the new SIRM President-elect.

Paraphrasing a historic phrase by Oscar Wilde, I feel like saying*: from next week I will no longer have to look for a job… nor will it have to look for me.*

Thank you all.

Andrea Giovagnoni,

Editor in-Chief,

La Radiologia Medica.

